# A commentary on ‘Updated disease distributions, risk factors, and trends of laryngeal cancer: a global analysis of cancer registries’

**DOI:** 10.1097/JS9.0000000000001355

**Published:** 2024-04-03

**Authors:** Zhongliang Fu, Jiajia Lv

**Affiliations:** a Department of Otorhinolaryngology, The People’s Hospital of Liaoning Province, Shenyang, Liaoning, People’s Republic of China; b Department of Otorhinolaryngology, The First Affiliated Hospital of China Medical University, Shenyang, Liaoning, People’s Republic of China

*Dear Editor,*

It was with great interest that we read the article published in the *International Journal of Surgery*. Huang *et al*.^1^ conducted a global review of cancer registries to determine the incidence and mortality rate of laryngeal cancer. According to research, the fact that overall global trends in laryngeal cancer have been decreasing, particularly among men, could be attributed to reduced tobacco and alcohol consumption.

Laryngeal carcinoma (LC) is the most frequent cancer in otolaryngology, accounting for 30–40% of head and neck cancers. The location and size of the initial tumor determine the wide range of clinical presentation associated with laryngeal cancer. A lump or pain that won’t go away, a sore throat, trouble swallowing, and hoarseness of voice are some symptoms. Early-stage laryngeal malignancies can be effectively treated with curative surgery, resulting in 80–95% local control rates; in contrast, regionally advanced larynx cancers are linked to substantially lower control rates, ranging from 40 to 70%. The survival rate can reach 80% in patients with locally isolated laryngeal cancers but drops to 46% in those whose tumors have spread to surrounding tissues and/or the regional lymph nodes and can reach 34% in patients whose cancer has already spread to other organs at the time of diagnosis. However, the survival rate may also depend on the staging and location of the cancer (subglottis, glottis, or supraglottis)^2^.

Smoking and excessive alcohol consumption are clearly linked to the development of upper aerodigestive tract squamous cell carcinomas. The American Cancer Society lists the following as the primary risk factors for LC: smoking, binge drinking, gastric reflux, Plummer-Vinson syndrome, anatomical anomalies, heat, chemicals, asbestos, nickel, or ionizing radiation exposure, as well as certain viral infections. According to some research, a patient’s gender can predict their prognosis independently for LC patients, with male patients having the lowest short-term and long-term survival rates^3^. However, a meta-analysis indicates that for individuals with laryngeal cancer, sex is not a major independent predictive factor^4^.

The purpose of this study was to examine the epidemiological trend of laryngeal cancer using the most recent data on the disease burden’s worldwide distribution. This novel approach provides insightful guidance and inspiration for the further development of this field of study. Nevertheless, there were several constraints. First, the COVID-19 pandemic’s impact was not factored into the GLOBOCAN 2020 forecasts, which were derived from historical data on incidence and mortality patterns^5^. Given that fewer cancer diagnoses were anticipated as a result of the pandemic, it’s possible that the incidence was overstated. Second, if registries in the nation’s capital or other important city were the source of the incidence and mortality data for that nation, there may have been an excess of reporting. Third, as health examinations are conducted more frequently in wealthy nations, more cases may be found.

It is necessary to upgrade the systems to keep track of and report new cases of LC. Because of poverty, which is linked to an increased risk of cancer, aging, and the presence of risk factors related to urbanization, the burden of LC rises in the absence of targeted action. It is possible to stop the increase in morbidity and death by encouraging healthy lifestyles and discouraging alcohol consumption.

## Ethical approval

This manuscript is a comment. Don’t need ethical approval.

## Consent

This manuscript is a comment. Don’t need patients consent.

## Sources of funding

Not applicable

## Author contribution

Z.F.: study concept or design, data collection, data analysis or interpretation, and writing the paper; J.L.: study concept or design and writing and revising the paper.

## Conflicts of interest disclosure

This manuscript is a comment without conflicts of interest.

## Research registration unique identifying number (UIN)

This manuscript is a comment. Don’t need UIN.

## Guarantor

Jiajia Lv.

## Data availability statement

This manuscript is a comment. Don’t need a Data availability statement. However, all the data from the current study are publicly available.

## Provenance and peer review

This manuscript is a comment without being invited.
